# Fiddler crab bioturbation determines consistent changes in bacterial communities across contrasting environmental conditions

**DOI:** 10.1038/s41598-019-40315-0

**Published:** 2019-03-06

**Authors:** Jenny Marie Booth, Marco Fusi, Ramona Marasco, Tumeka Mbobo, Daniele Daffonchio

**Affiliations:** King Abdullah University of Science and Technology (KAUST), Red Sea Research Center, Thuwal, 23955-6900 Saudi Arabia

## Abstract

Ecosystem functions are regulated by compositional and functional traits of bacterial communities, shaped by stochastic and deterministic processes. Biogeographical studies have revealed microbial community taxonomy in a given ecosystem to change alongside varying environmental characteristics. Considering that stable functional traits are essential for community stability, we hypothesize that contrasting environmental conditions affect microbial taxonomy rather than function in a model system, testing this in three geographically distinct mangrove forests subjected to intense animal bioturbation (a shared deterministic force). Using a metabarcoding approach combined with sediment microprofiling and biochemistry, we examined vertical and radial sediment profiles of burrows belonging to the pantropical fiddler crab (subfamily *Gelasiminae*) in three contrasting mangrove environments across a broad latitudinal range (total samples = 432). Each mangrove was environmentally distinct, reflected in taxonomically different bacterial communities, but communities consistently displayed the same spatial stratification (a halo effect) around the burrow which invariably determined the retention of similar inferred functional community traits independent of the local environment.

## Introduction

Biogeography of microorganisms is determined largely by stochastic processes^1–3^, but deterministic processes, essentially related to their ecological niche, are now known to play a significant role in shaping community composition in a given system^4–6^. The unsurmountable importance of microorganisms as the drivers of global biochemical cycles^7^ is reflected in their broad taxonomic and metabolic functional diversity^8^. For many of their diverse metabolic functions, microbial communities exhibit functional redundancy, whereby different taxa are able to perform the same metabolic function^9–11^, for example nitrogen cycling in soil^12^ and methanogenesis in bioreactors^13^. For this reason, more recent studies of microbial community structure have tended to focus on functional structure rather than taxonomic structure, with a general consensus of the decoupling of some metabolic functions with taxonomic composition in various environments^14,15^, i.e., the conditions of the environment hold more weight in shaping functional group distribution than that of taxonomic composition.

Microbial community traits have been compared from the local to continental scale with diversity in both taxonomy and functionality being attributed, across the range of spatial scales, to native environmental conditions^8^. However, due to constraints such as the availability of electron acceptors and heterogeneity in, for example, pH, temperature and salinity^16^, environmental drivers do not explain well the variation in taxonomic composition observed in systems with similar environmental conditions: a pattern which has been observed across many marine and terrestrial systems^10,12,17^. In a system with the same physico-biochemical features (bromeliad aquatic plants), Louca *et al*.^10^ observed intersystem taxonomic, but not functional, variations in the microbial communities that were not completely explained by the different environmental characteristics. Recently, variation in oceanic environmental conditions was described to be responsible for structuring the function of the marine microbial community, while these conditions were only able to weakly explain taxonomic variation within functional groups^15^.

Ecological resilience, and the capacity of a system to adapt to change, is positively influenced by higher functional community diversification, such as denitrifiers or carbon degraders, and increased taxonomic diversification within these functional groups^18,19^. Here we hypothesize that in the same model system under contrasting local environmental conditions, such as those occurring across large biogeographical ranges, broad and fine scale microbial functional and interaction patterns, rather than taxonomy, should be conserved around a consistent source of selective pressure.

To test this hypothesis, we studied mangrove sediments subjected to the deterministic selective pressure of intense animal bioturbation, a process known to enhance biological activity and modify physical and chemical properties of sediment^20–22^. Mangrove forests are sites of strong environmental selection due to the intrinsic characteristics of intertidal environments and the prevalence of bioturbating organisms that, through the creation of burrows, impose a selective pressure at the interface of aquatic and terrestrial habitats^23^ associated with microbial hotspots^24^. Thus, mangrove sediment is an ideal system to explore spatial changes in microbial community traits. Using a fiddler crab burrow as our model, we extended our study over a broad geographical (latitudinal) range to encompass contrasting environments.

## Results

**Figure 1:**
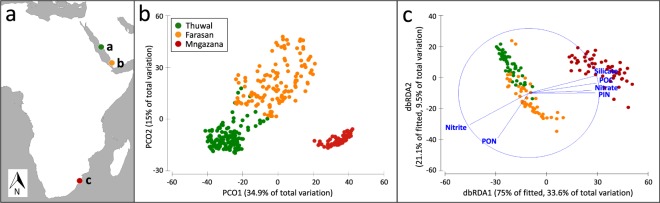
Study site variation. (**a)** Map of study sites: a - Thuwal, b - Farasan, c - Mngazana; **(b)** Principal coordinates analysis of total bacterial OTU assemblages categorized by site (n = 384); **(c)** Distance-based redundancy analysis (db-RDA) showing significant biogeochemical drivers of bacterial community composition at each site.

Our geographic range encompassed the sites Thuwal, Farasan and Mngazana (Fig. 1a), in which we radially sampled sediment ‘Fractions’ around burrows and in surface, subsurface and deep sediment. The mangrove stands in Thuwal, Farasan and Mngazana displayed diverse sediment characteristics in terms of biogeochemistry, metal content and grain size (Supplementary Fig. S1, Supplementary Table S1 and Supplementary File S2). Accordingly, PCoA of bacterial OTU composition segregated the three geographical sites into distinct groups, accounting for 49.9% of dissimilarity in community composition between sites (Fig. 1b). A significant effect of ‘Site’ (*P* = 0.0001) and ‘Burrow’ (*P* = 0.0138) was observed on biogeochemistry (PERMANOVA, Supplementary Table S1). Sulphate, nitrite and nitrate contributed 76.4% to dissimilarity between Farasan and Thuwal sediment (SIMPER), with nitrite and nitrate being higher in Farasan and sulphate being higher in Thuwal. POC, PON, phosphate and silicate contributed 55.6% to dissimilarity between Mngazana and Thuwal (SIMPER), with the former being more abundant in Thuwal, while PON, phosphate and silicate were more abundant in Mngazana. PIC, phosphate, PON and silicate contributed 67.2% to dissimilarity between Mngazana and Farasan (SIMPER), with PIC being more abundant in Farasan, and phosphate, PON and silicate being more abundant in Mngazana.

Site-specific bacterial assemblages correlated with site-specific physico-chemical characteristics (Fig. 1c, Supplementary Fig. S2, Supplementary Table S2). Across the three mangrove forests, POC, PON, PIN, nitrate, nitrite and silicate significantly explained bacterial community variability amongst sites (Fig. 1c, DistLM, AICc = 1128.9, R^2^ = 0.44).

**Figure 2:**
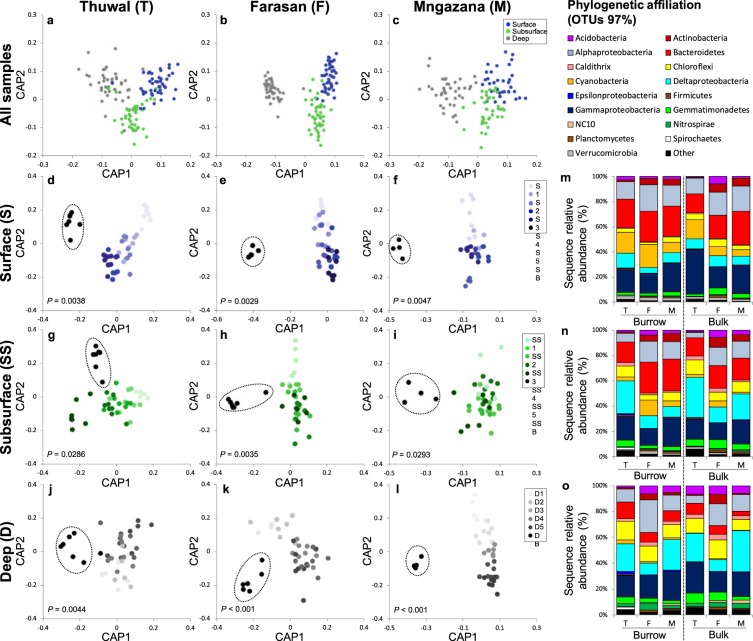
OTU variation amongst sediment fractions. Canonical Analysis of Principal coordinates (CAP) of OTU abundance in surface, subsurface and deep sediment for Thuwal **(a)**, Farasan **(b)** and Mngazana **(c)**. Canonical Analysis of Principal coordinates (CAP) of the bacterial OTU abundance at each ‘Site’, ‘Depth’ and ‘Fraction’ **(d–l)**. Taxonomy bar charts show the relative contribution of different taxa to overall bacterial community composition in burrow and bulk sediment in Thuwal, Farasan and Mngazana in **(m)** surface, **(n)** subsurface and **(o)** deep sediment.

A significant ‘Site’ × ‘Depth’ × ‘Burrow’ interaction was observed on bacterial OTU assembly; at each site, bacterial communities in surface, subsurface and deep sediment displayed significantly different OTU composition amongst depths (GLM, df = 4,366, Dev = 17397, *P* = 0.014; Fig. 2a–c). Comparison of bacterial composition of the different sediment fractions at different depths revealed a significant effect of ‘Fraction’ across all sites at all depths, with bulk sediment consistently segregating from burrow sediment (*P* < 0.05 in all cases; Fig. 2d–l, Supplementary Table S3).

The factor ‘Site’ explained up to 42% of the entire bacterial betadiversity variability. However, at each site the ‘Depth’ accounted for 11% (Thuwal), 25% (Farasan) and 24% (Mngazana) of variability. The variability explained by ‘Burrow’ accounted for 4% (Thuwal), 3% (Farasan) and 4% (Mngazana) of total variability.

‘Site’ and ‘Burrow’ significantly affected OTU alpha diversity (Shannon Index; PERMANOVA, F_2,362_ = 17.54, *P* = 0.0001 and F_1,362_ = 6.4, *P* = 0.015, respectively) and richness (Species richness index; PERMANOVA F_2,362_ = 20.83, *P* = 0.0001 and F_1,362_ = 12.82, *P = *0.0005, respectively). Mngazana had higher diversity and richness (H’ ± SE: 5.63 ± 0.03 and S ± SE: 91.1 ± 1.1, respectively) than both Farasan (5.39 ± 0.05 and 84.7 ± 1.8, respectively) and Thuwal (4.98 ± 0.1 and 71.9 ± 2.3, respectively). Bulk sediment displayed lower values for both parameters compared to burrow sediment, while no effect of ‘Depth’ on either diversity or richness was observed (*P* > 0.05).

Bacterial communities across sites, depths and fractions were comprised of the same dominant phyla with different overall contributions (Supplementary Fig. S3). Site-specific patterns in community composition were observed (Fig. 2m–o). Farasan and Mngazana had a larger contribution of *Actinobacteria* (1% to 7%) and *Acidobacteria* (1% to 6%) in surface and subsurface sediment compared to Thuwal (0.1% to 1% and 0.1% to 3%, respectively). Mngazana had a reduced contribution of *Cyanobacteria* to community composition compared to Thuwal and Farasan. Thuwal also had a larger contribution of *Spirochaetes* and a smaller contribution of *Planctomycetes* compared to other sites. Notably, Farasan had a smaller contribution of *Deltaproteobacteria* and larger contribution of *Alphaproteobacteria* compared to Thuwal and Mngazana. Furthermore, although only a small contribution of *Epsilonproteobacteria* and *Betaprotobacteria* was observed across sites, they had a greater contribution to the overall community in Thuwal and Mngazana compared to Farasan. Rare OTUs (with a contribution of less than 1% to total community composition) had a greater overall contribution in Thuwal than the other sites (up to 5.9%).

Differences between burrow and bulk sediment were observed at all sites (Fig. 2m–o). Generally, burrow sediment had a higher contribution of *Cyanobacteria*, *Verrucomicrobia*, *Spirochaetes* and *Bacteroidetes* than bulk sediment, whereas bulk sediment had a higher contribution of *Delta-* and *Gammaproteobacteria*. Thuwal and Farasan had a consistently smaller *Alphaproteobacteria* component in bulk sediment compared to burrow sediment across all depths.

Ternary plot analysis revealed an overall higher density of OTUs in burrows compared to their bulk sediment counterparts (Supplementary Fig. S4). Notably, more OTUs were shared between the surface and deep in the burrow compared to bulk sediment at all sites, and Thuwal in particular displayed the same degree of shared OTUs between these two depths. Thuwal bulk sediment displayed the least sharing of OTUs between depth levels. Farasan had very few OTUs shared between the surface and deep in either the burrow or bulk sediment. At Mngazana, no OTUs were exclusively enriched in the subsurface, either in the burrow or bulk, but a large number of OTUs were unique to the deep.

Bulk and burrow sediment communities hosted discriminant taxa at each site (LDA, LEfSe; Supplementary File S3). A greater number of significantly differential taxa between bulk and burrow sediment were observed in Thuwal compared to the other mangroves (Supplementary Fig. S5). In Thuwal, *Proteobacteria*, *Chloroflexi* and *Actinobacteria* contributed the greatest proportion of differentially abundant OTUs in burrow sediment. In Farasan, the phyla *Planctomycetes* and SAR406 were discriminately more abundant in burrow than bulk sediment, and a large contribution of phyla from *Proteobacteria*, *Chloroflexi*, *Acidobacteria* and *Actinobacteria* were differentially more abundant in bulk sediment than burrow (Supplementary Fig. S5). In Mngazana, *Actinobacteria*, TM7 and *Verrucomicrobia* were differentially more abundant in burrow than bulk sediment, and *Proteobacteria* and GNO4 phyla were more abundant in bulk than burrow sediment (Supplementary Fig. S5). A large contribution of sulphate-reducing bacteria from *Deltaproteobacteria*, *Firmicutes* and *Nitrospirae* to the overall bacterial community, being more abundant in bulk sediment, was observed (LDA effect sizes; Supplementary File S3). We detected three species of known cable bacteria belonging to the family *Desulfobulbaceae*: *Desulfopila aestuarii*, *Desulfobulbus mediterraneu*s and *Desulfobulbus rhabdoformis* (>97% similarity), with differential abundances at the three sites. At Mngazana, *Desulfopila aestuarii* was the most abundant of the three species, while *Desulfobulbus mediterraneu*s was the most abundant at both Thuwal and Farasan.

**Figure 3:**
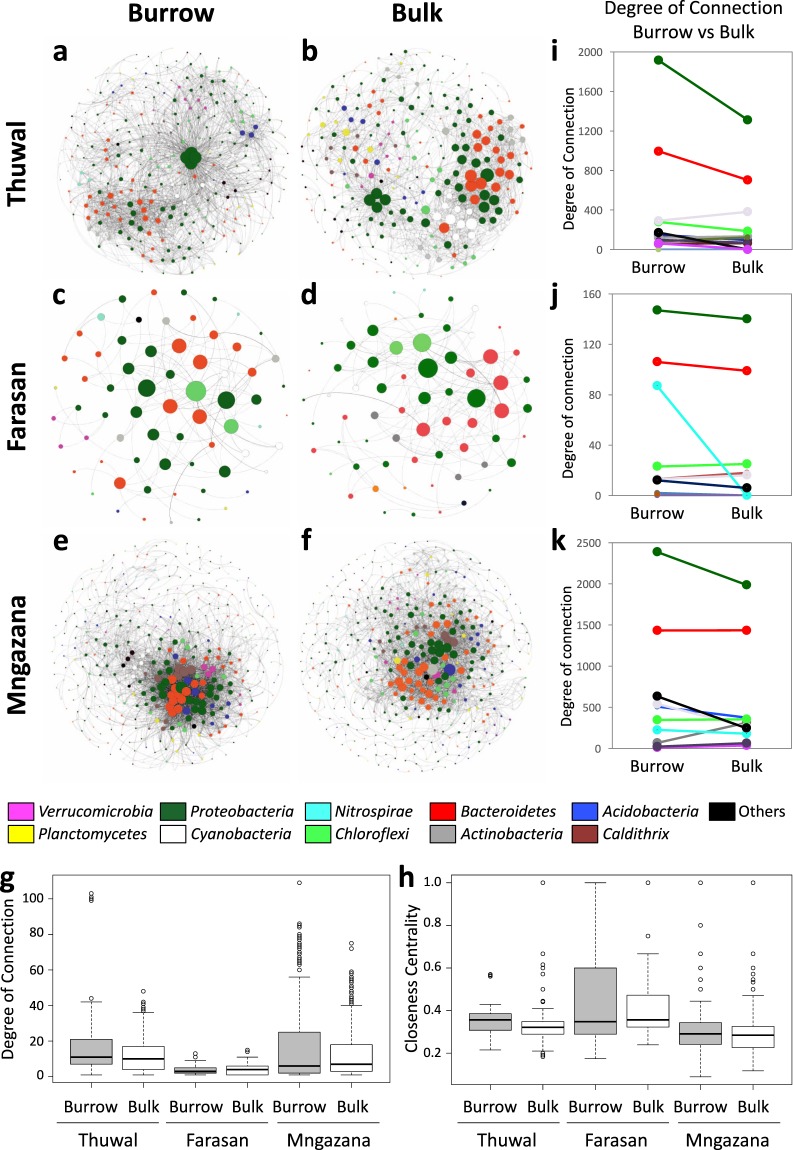
Co-occurrence network analysis of bacterial OTUs in burrow and bulk sediment. Interaction among OTUs in bulk and burrow sediment at **(a**,**b)** Thuwal, **(c**,**d)** Farasan and **(e**,**f)** Mngazana. Boxplots represent the overall degree of connection **(g)** and closeness centrality **(h)** of the nodes of each network. **(i–k)** Phylum degree of connection in burrow and bulk sediment at each site. In each network, the nodes correspond to the OTUs present and are coloured according to phylum affiliation (>97%).

Network co-occurrence analysis revealed a significantly different structure between burrow and bulk sediment at each site (Fig. 3 and Supplementary File S4). Over centrality parameters significantly varied for ‘Site’ (degree of connection: GLM, Chi-square = 124.78, d.f = 2, *P* < 0.0001, Fig. 3g; closeness centrality: GLM, Chi-square = 124.78; d.f = 2; *P* < 0.0001, Fig. 3h). A significant variation in the connectivity of *Proteobacteria* and *Bacteroidetes*, consistently decreasing in bulk sediment in all three sites was observed, while for the other taxa there were site-specific variations (Fig. 3i–k). *Proteobacteria* and *Bacteroidetes* had a higher degree of connection in Thuwal and Mngazana, and the clustering coefficient was also higher at these two sites, compared to Farasan (Supplementary Table S4). The number of interactions was highest in Mngazana (burrow: 3039, bulk: 2655) and lowest in Farasan (burrow: 134, bulk: 153) and modularity was higher in Thuwal than other sites. The clustering coefficient was higher, and equal, in burrow sediment in Thuwal and Mngazana (0.34) than in bulk sediment (0.28 and 0.11, respectively), while Farasan had the lowest values. Network centralization was also much lower in Farasan than the other two sites. Co-presence interactions were consistently higher than mutual exclusion interactions in both bulk and burrow sediment at each site.

Contrary to bacterial community composition, no significant interaction of ‘Site × Depth × Burrow’ on predicted metabolic function (*P* > 0.05), but instead a main effect of ‘Site’, ‘Depth’ and ‘Burrow’, was observed (Supplementary Table S5 and Supplementary Fig. S6). Functions significantly differed according with ‘Site’, for example denitrification was more prevalent in the South African mangrove, while nitrogen fixation and cyanobacteria were more abundant in the Red Sea mangroves. Other functions, instead, were consistently conserved across all study mangroves relative to bioturbation; for example, bacteria performing phototrophy, anoxygenic photoautotrophy and chemoheterotrophy were consistently higher in the burrow than in the bulk sediment in each mangrove.

**Figure 4:**
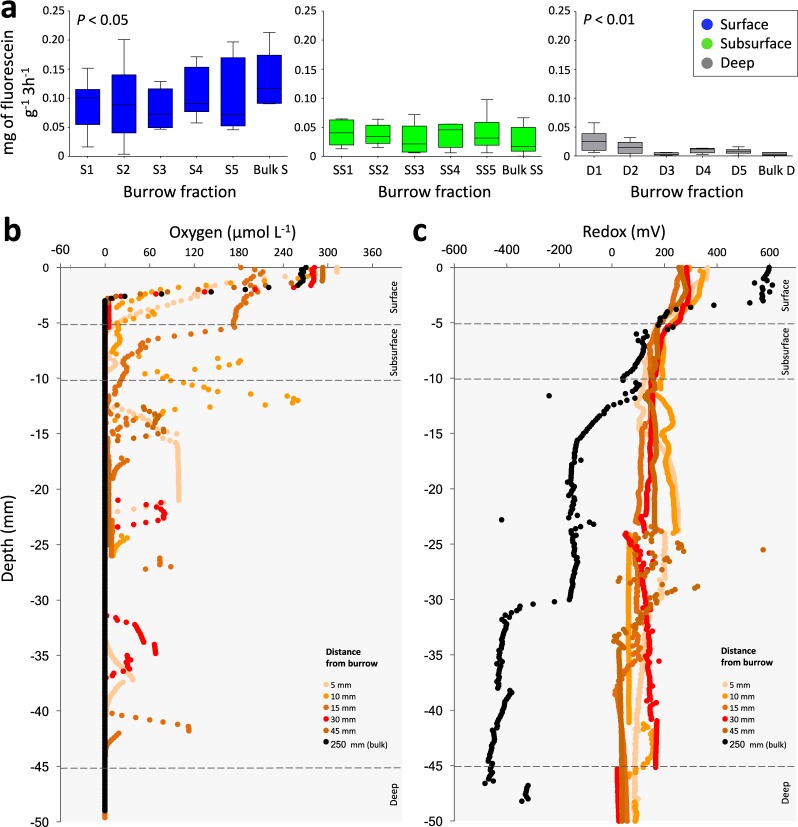
Sediment microbial activity, oxygen concentration and redox. (**a)** Fluorescein diacetate analysis. Boxplots with standard error of mean showing amount of Fluorescein produced by microbial activity for each sediment fraction in surface, subsurface and deep sediment at Thuwal (n = 6 for each fraction at each depth level). **(b)** Oxygen concentration microprofiles and **(c)** redox microprofiles of Thuwal burrow and bulk sediment along a gradient from the burrow wall to bulk. N = 3 for each replicated distance from burrow wall.

Microbial activity, measured in Thuwal, consistently decreased from surface to deep sediment across all burrow fractions and in bulk sediment (Fig. 4a). A significant effect of ‘Fraction’ was observed only in the surface and deep sediment; microbial activity increased towards bulk sediment in the surface (ANOVA, F_5,30_ = 2.598, *P* < 0.05), while the opposite trend was observed in deep sediment (ANOVA, F_5,30_ = 5.056, *P* < 0.01).

In Thuwal, oxygen concentrations around the burrow decreased from approximately 300 µmol L^−1^ to 0 µmol L^−1^ at approximately 5 mm depth in all sections of the burrow wall and bulk sediment (Fig. 4b). Interestingly, random pulses of oxygen between 10 and 40 mm depth were consistently recorded in fractions 1–5 around the burrows, but not in the bulk sediment. Redox potential profiles differed between the burrow fractions and the bulk sediment. In fractions 1–5 around the burrow redox was always positive (between 0 and more than 200 mV) and comparatively stable down to depths of more than 40 mm; while it progressively decreased in the bulk sediment to negative values (around −200 mV) already below 15 mm depth, reaching −400 mV below 30 mm (Fig. 4c).

## Discussion

We show that, despite the main drivers of geographic location and depth, bioturbation creates a fine spatial scale selective pressure that determines a significant consistent rearrangement of sediment bacteria community assemblages and interactions along a burrow-induced gradient. These patterns invariably occur across a broad geographical scale in mangroves with contrasting ecology and sediment biochemistry. Sediment bacterial functional rearrangements were independent of the biogeographical community taxonomy differences and those in biogeochemistry at each site and highlight a functionally-conserved altering effect of the burrows under various contrasting sediment environmental conditions.

Taxonomic composition, expectedly, differed across mangroves, reflected in differential functional community composition, e.g. *Cyanobacteria* were more abundant in the Red Sea mangroves, whereas denitrifying bacteria were more abundant in the South African mangroves in accordance with a higher input of nitrogen. However, while we detected a significant effect of the interaction of geographic location, depth and sediment type (i.e. burrow vs. bulk) on the taxonomic composition and network interactions of the bacterial communities in the mangroves studied, in terms of functional structure this interaction was not significant. Despite the differences imposed by diverse physico-chemical characteristics in terms of function, we measured a consistent effect of the burrow halo on metabolic function across geographic locations, regardless of the specific function, with certain functions being consistently more abundant in burrow sediment compared to bulk sediment such as phototrophy, anoxygenic photoautotrophy and chemoheterotrophy. While this type of analysis provides only broad functional resolution and cannot resolve the functional complexity, this approach is able to broadly screen potential functions of the bacterial community and adds another dimension to previous studies of macrofaunal burrowing effects in intertidal sediments, such as polychaetes^25^ and shrimps^26^. Undoubtedly macrofaunal bioturbation stimulates bacterial activity and increases the number of heterotrophic niches available for bacteria^27^.

The bacterial community composition of the South African mangroves was particularly affected by POC, PON, PIN, nitrate, silicate and iron (all particularly high in sediment compared to the Red Sea mangroves), explained by the high input of nutrients and metals in the riverine setting and the absence of run-off and other freshwater inputs in the Saudi Arabian fringing mangroves. Nitrogen fixation and denitrification processes were enhanced in the South African mangroves mainly due to riverine input of nitrogen, particularly enriched in rural areas^28^. Mangroves in the Red Sea instead are nutrient limited^29^ and the only nitrogen input results from bacterial activity enhanced by the tide and the cyanobacterial mats that can fuel nitrogen fixation and therefore nitrogen input to the system^30^.

Cuellar-Gempeler and Leibold^31^ recently showed that multiple colonist pools exist in fiddler crab burrows in intertidal sediment, corroborating the diverse sediment environment conditions at different depths and diverse selection pressures detected in this study. Similar to the rhizosphere, where changes in soil microbial community structure and complexity are mediated by the plant root effect^32^, burrows influence the overall composition of the surrounding sediment microbiome enhancing network complexity. While the community assemblage is a defining characteristic of the burrow, so too is the network complexity, and a greater proportion of co-presence interactions in the burrow than in the bulk sediment suggests the creation of microniches that can support a more connected bacterial community. Although beyond the scope of this study, bacteria dynamically interact with other sediment microorganisms, namely archaea and fungi, to form complex networks responsible for controlling organic matter decomposition and nutrient availability^33^. For example, in the mangrove sediments in our study it is likely that methanogenic archaea and sulphate-reducing bacteria form syntrophic communities together to degrade organic matter^34^. Interestingly, the bacterial network properties of sediment were similar for Thuwal and Mngazana, but not for Farasan that had a poorly connected sediment bacterial community. Indeed, this finding is also supported by a reduced number of shared OTUs at all depths in Farasan sediment, which may be attributable to the peculiar environmental setting of fossil coral bedrock. The sediment derived from fossil corals is known to be nutrient-poor with comparatively few microorganisms^35^.

Sediment oxygen content dropped rapidly to zero at approximately 5 mm depth, as previously observed in some mangrove sediments^36^. Unlike many marine animals that actively irrigate their burrows during high tide (e.g. polychaetes, shrimps and bivalves), fiddler crabs plug their burrows to allow air-breathing at high tide^37^, thus trapping air and creating aerobic microniches along the burrow walls that are retained throughout submersion (Supplementary File S5). Despite continuous burrow exposure to air, we did not observe any substantial augmentation of oxygen concentration through the burrow wall. We did, however, observe pulses of oxygen, probably due to the presence of infaunal burrowers that exploit the main crab burrow for shelter and dig small tunnels through the walls^38^, often reaching the same concentration as surface sediment in bioturbated sediment which were absent in bulk sediment. This immediately challenges the concept that mangrove sediment is highly anoxic, particularly if we consider the high abundance of burrows and roots in mangroves that reach depths much greater than those sampled in this study^39^. Oxygen is rapidly consumed by microbial communities below the sediment surface^40^ and burrows essentially extend the oxic/anoxic interface, producing a millimetre-thin layer of oxygenated sediment at depth which creates a zone of increased biogeochemical reactions and microbial activity^41^. Indeed, we observed that the microbial activity in the deep was highest at the burrow wall and decreased toward bulk sediment, which is further supported by increased CO_2_ efflux rate from crab burrows compared to surrounding sediment^42^. Not only does the oxic zone affect the distribution of aerobic and anaerobic taxa^43^, but it has an overall cascade effect on the bacterial respiration pathways and community assemblages^41^. The oxidizing effect of the burrow affects sediment redox potential and we observed this feature to be consistently positive to at least 40 mm depth and negative in bulk sediment (around −200 mV below 15 mm depth, dropping to −400 mV below 30 mm depth), which is in accordance with previous studies of fiddler crab burrows^44^. The unsteady-state redox of mangrove sediment was highlighted in a recent study of mangrove sediment cores which presented evidence that oxygen input causes sudden and significant reoxidation of reduced sulphur^45^.

Sulphate-reduction is a major respiration pathway in anaerobic mangrove sediment, which accounts for almost 100% of sediment CO_2_ emission in some mangrove systems^46^. Mangrove sediment sulphate-reducing bacteria are diverse^36^ and in this study we detected more abundant and diverse sulphate-reducing taxa (i.e., *Deltaproteobacteria* and *Nitrospirae*) in the bulk than the burrow sediment. This accords with our recorded absence of oxygen below the surface in bulk sediment. Indeed, the importance of bioturbation in sulphate reduction rates has been highlighted^47^, showing effective prevention of sulphide accumulation in mangrove sediment bioturbated by fiddler crabs due to sulphide reoxidation^48^. In Mngazana and Farasan mangroves, members of the family *Desulfobulbaceae* may have important ecological roles in sediment sulphide oxidation. Notably, active cable bacteria belonging to this family have been recorded in mangrove sediment^49^. Due to their high filament densities, this group is responsible for long-distance transport of electrons in deep sediment to the surface that are harvested by sulphide oxidation^50^. Bioturbation has been suggested to constrain the distribution of this group due to the cutting of filaments during sediment reworking^51^, which may explain the discriminant abundance of this group in bulk sediment.

The burrow effect that we describe above is more complex than the effect of the structure itself, and it is essential to consider the ecology of the burrow host. Fiddler crabs have a strict fidelity to their burrows and thus perform all of their activities, including surface grazing, within a few centimetre radius of their burrow^52^. Organic content has been shown to be the strongest physical sediment characteristic affecting where fiddler crabs forage, linked to the higher abundance of microorganisms associated with these patches^53^, and accordingly we recorded a halo of reduced bacterial activity in grazed surface sediment. Sediment is continuously reworked by fiddler crabs during burrow maintenance, bringing pellets from inside the burrow to the surface (excavation pellets; Supplementary File S6). Indeed, in all sites we observed that a large number of OTUs were shared between the surface and the deep burrow sediment, which was absent in bulk sediment. We also observed that *Cyanobacteria* had a comparatively large contribution to community composition in the deep at burrow walls, indicating transport of surface sediment to the deep. This sediment “mixing” is likely to be one of the main factors responsible for the differences between burrow and bulk sediment we observed in this study.

To comprehend the impact of burrowing on the mangrove sediment environment, in terms of the modification of both taxonomic and functional diversity, we considered the density of burrows by focusing on those of fiddler crabs. Each burrow was a 1 cm × 5 cm void in the sediment, and we determined a 10 cm diameter halo of influence with an area of 78.5 cm^2^ for one burrow. Based on our estimates, at a density of 25 to 41 burrows per m^2^, this equates to an area of fiddler crab burrow influence ranging from 1, 962 to 3, 218.5 cm^2^ per m^2^ of sediment to a depth of 5 cm. If we extend this to an ecosystem scale, this influence accounts for approximately 20 to 35% of mangrove sediment. In Kenyan mangroves, densities of fiddler crabs up to 100 per m^2^ have been reported^54^, which raises this percentage of sediment to 78.5% of the total mangrove extension. Consequently, their burrows are imposing selective pressure on a large portion of mangrove sediment. Furthermore, this is surely an underestimation because this calculation is exclusively restricted to fiddler crab burrows, of which we did not investigate the entire burrow shaft (reaching up to 20 cm of depth). Bioturbation by other crab species and other animals including ants, shrimps and mudskippers is also extensive in mangroves and contribute to form more complex and deeper burrow structures^55^. We can therefore predict that the described bioturbation effect has a large overall impact on the mangrove ecosystem, by altering the nature of the sediment microbiome, which ultimately governs environmental processes, such as carbon and nitrogen fluxes, in this coastal ecosystem.

## Conclusions

**Figure 5:**
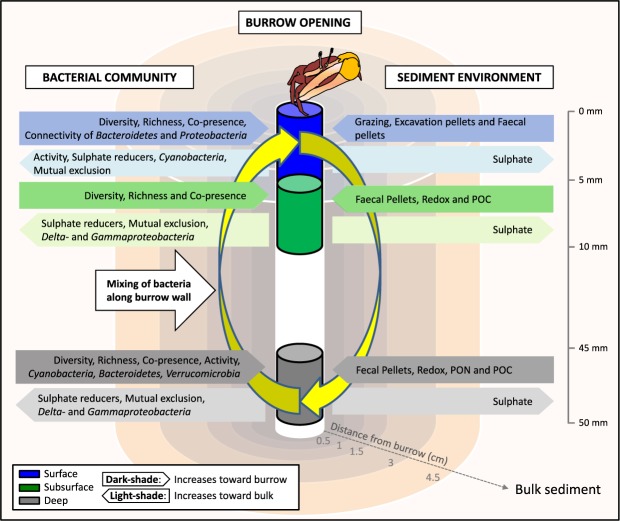
Generalised conceptual model of the burrow halo. The overall effects of the burrow on the sediment environment and bacterial community are displayed on either side of the burrow model. Blue, green and grey represent the surface, subsurface and deep sediment layers, respectively. Within each layer, the dark-shade indicates features of the bacterial community or sediment environment that increase toward the burrow; while the light-shade indicates the increase of these features toward bulk sediment. The mixing of sediment along the burrow funnel due to crab activity is indicted.

Here we demonstrate that macrofaunal burrows in mangrove sediment apply a consistent radially-distributed selective pressure, a halo effect, under contrasting physico-chemical conditions, across a broad latitudinal range. This halo effect controls the diversity, interactions and function of sediment microbial assemblages (Fig. 5). While taxonomic community structure was not retained across the large geographical range we examined, due to the local diverse environmental conditions, the selective pressure of the burrow invariably determines the retention of similar functional community traits independently of the local environment. This study is a baseline for further investigation of the role of sediment microorganisms in the overall functioning of the ecosystem, highlighting the necessity for a fingerprint to footprint approach^56^. Crab burrows act as a hotspot of diversity and functionality that can increase ecological resilience through functional redundancy and we believe these structures can be of pivotal interest in restoration and rehabilitation projects^57^. However, we highlight the need to include other components of the microbiome, namely archaea, fungi and microeukaryota^58,59^, and also other components of the ecosystem such as the burrow host^60^, whose interactions can boost and modulate the entire sediment biological function.

## Methods

### Study sites and sampling

Sampling was performed across a large latitudinal range in three mangrove stands: the middle of the Red Sea (Thuwal, 22°33′N 41°24′E, April 2016), nearby the Aden gulf where the Red Sea enters the West Indian Ocean (WIO) (Farasan Island, 16°20′N 41°24′E, March 2015) and at the southern extension of mangrove distribution in the WIO in South Africa (Mngazana, 31°42′S 29°25E, April 2016) (Fig. 1a). The mangroves in each study site have contrasting characteristics, in terms of temperature, precipitation, tidal range, geomorphological setting and floral composition (Supplementary Table S6).

Sampling was carried out following the same design at each site at low tide, when burrows were uncovered, during the period of Spring Tide. Burrow density was assessed at each site, within the zone occupied by fiddler crabs, one of the dominant bioturbators in each mangrove^61^ (Supplementary Fig. S7), considering burrows measuring 10 mm diameter (mean burrow density per m^2^ ± Standard Error (SE): Mngazana 42 ± 5, Farasan: 32 ± 2 and Thuwal: 25 ± 4). Eight active burrows belonging to male crabs were selected randomly along a 200 m long transect, being suitable only if they were more than 30 cm from another burrow, plant or pneumatophore and collecting a total of 18 samples per burrow (15 burrow, 3 bulk) for a total of 144 samples per site. The design incorporated radial sampling^40^ from the burrow wall to 4.5 cm distance at three depth levels: surface (0–0.5 cm), subsurface (0.5–1.0 cm) and deep (5–5.5 cm) (Supplementary Fig. S8). Bulk sediment was sampled at the three depths at a distance >30 cm away from another source of bioturbation. Taking into consideration the non-vertical nature of fiddler crab burrows down towards the burrow chamber^55^, we restricted our sampling to the upper portion of the burrow and verified their verticality through the fine-scale dissection method of sediment sampling we adopted. For each burrow sampled, sediment was collected for DNA extraction and biogeochemical analysis and stored at −20 °C, while sediment for microbial activity analysis was processed in the laboratory within 30 min of sampling.

Biogeochemical, metal and grain size analyses were performed in GEOMAR (Kiel, Germany) following established protocols detailed in Supplementary File 1.

DNA was extracted from a 0.4 g sub-sample of each 432 sediment samples and the V4-V5 hypervariable regions of the 16S rRNA gene were amplified by PCR using specific primers (341F, 785R). Library preparation was carried out with the 96 Nextera XT Index Kit (Illumina^®^) following the manufacturer’s instructions. PCR products were sequenced using the Illumina^®^ MiSeq platform with pair-end sequencing at the Bioscience Corelab, King Abdullah University of Science and Technology. Paired end reads measured an average 500 bp in length. Details of raw data processing are provided in Supplementary File S1.

Fluorescein diacetate (FDA) hydrolysis assay was used to assess total microbial hydrolysing activity in sediment^62^ in the Thuwal mangrove; the amount of fluorescein released from each sediment sample was calculated referring to a standard curve (see Supplementary File S1).

Oxygen and redox (Eh) were measured with microsensors (Ox-200 and Redox-200 microelectrodes with a tip diameter of 200 μm, UNISENSE, Aarhus, Denmark) in sediment cores extracted at low tide (during daylight) in the Thuwal mangrove. Sediment cores were taken around a central crab burrow using PVC cores (diameter 15 cm) and bulk sediment cores were taken in unbioturbated sediment. Microsensors performed vertical measurements (at a resolution of 200 μm) into sediment cores at interval distances away from the burrow wall (0.5, 1, 1.5, 3 and 4.5 cm; following the experimental design) and to a depth of 5 cm. Microsensor signals were recorded directly using the SensorsTrace Suite software (Unisense). Further details are provided in Supplementary File S1.

### Statistical analysis

#### Biogeochemical, metal and grain size analysis

Prior to analyses, variables with high multi-collinearity (correlation coefficient > 0.85) were removed, retaining: particulate organic carbon (POC), particulate organic nitrogen (PON), particulate inorganic nitrogen (PIN), particulate inorganic carbon (PIC), nitrate, silicate, phosphate and sulphate (biogeochemical) and iron (Fe), lead (Pb) and uranium (U) (metals). Homogeneity of multivariate dispersion was verified for each factor with the distant-based test (PERMDISP) and 3-way PERMANOVA (9999 permutations, Euclidean distance) was used to test differences in biogeochemistry, metal content and grain size amongst the factors (fixed, orthogonal) ‘Site’ (3 levels: Thuwal, Farasan, Mngazana), ‘Depth’ (3 levels: surface, subsurface, deep) and ‘Burrow’ (2 levels: burrow, bulk). SIMPER analysis determined which variables contributed most to variation in biogeochemistry, metal content and grain size within each ‘Site’. Grain size frequencies were analysed using R package “G2sd”. Differences in sediment phi (continuous response variable) were tested among the Factor ‘Fraction’ (6 levels: 1, 2, 3, 4, 5, bulk; Supplementary Fig. S8) for each ‘Site’ and ‘Depth’ using the “aov” function in R.

Distance-based multivariate analysis for a linear model (DistLM) was used to determine the biogeochemical variables, metals and grain sizes responsible for explaining community composition variation amongst sites with significance provided by the corrected Akaike information criterion (AICc)^63^.

#### Bacterial function analysis

The FAPROTAX database was used to assign bacterial OTUs to known metabolic or ecological functions http://www.zoology.ubc.ca/louca/FAPROTAX15. Nine of the most abundant and representative functions were selected for further analysis: aerobic nitrite oxidation, denitrification, nitrogen fixation, cyanobacteria, anoxygenic photoautotrophy, oxygenic photoautotrophy, photoheterotrophy, phototrophy and chemoheterotrophy.

#### Bacterial community and diversity analysis

Principal Coordinates Analysis (PCoA) using a Bray-Curtis dissimilarity matrix, was used to visualize the diversity in OTU abundance between sites. Differences amongst samples were tested using a multivariate generalized linear model (GLM) with a negative, bimodal error distribution in the R package “mvabund”, considering OTU as the multivariate response variable and the categorical factors (fixed, orthogonal) ‘Site’ (3 levels: Thuwal, Farasan, Mngazana), ‘Depth’ (3 levels: surface, subsurface, deep) and ‘Burrow’ (2 levels: burrow, bulk) as explanatory variables. Changes in bacterial community composition between sediment fractions (factor ‘Fraction’ fixed and orthogonal; 6 levels: 1, 2, 3, 4, 5 and bulk, respectively indicating distance from the burrow wall of 0.5, 1.0, 1.5, 3.0, 4.5 and >30 cm as shown in Supplementary Fig. S8) were tested using Canonical Analysis of Principal coordinates (CAP).

Ternary plots were created based on the mean relative abundance of OTUs in burrow and bulk sediment in each site using the R package “ggtern”. Linear discriminant analysis effect size (LEfSe, www.huttenhower.sph.harvard.edu/galaxy/) was performed to identify bacterial taxa discriminately more abundant in the bulk and burrow sediment at each site (Wilcoxon *P* value: 0.05, LDA > 2). Shannon diversity Index and OTU richness differences were tested with a 3-way PERMANOVA (calculated from the Shannon diversity Index and OTU richness) among the factors ‘Site’ (levels: Thuwal, Farasan, Mngazana), ‘Depth’ (levels: surface, subsurface, deep) and ‘Burrow’ (levels: burrow, bulk). With the same experimental design and test, we compared community functions computed with FAPROTAX, after checking homogeneity of the dispersion (PERMDISP, F_17,366_ = 0.45, *P* = 0.064).

A co-occurrence network was built using the routine CoNet in Cytoscape 3.2.1 to search for significantly co-existing or mutually exclusive OTUs between burrow and bulk sediment among the three sites. After removal of rare OTUs (less than 0.1% of sequences per sample), the network was constructed using a combination of the Pearson and Spearman correlation coefficients and the Bray-Curtis (BC) and Kullback-Leibler (KLD) dissimilarity indices. In order to calculate statistical significance of co-occurrence/mutual exclusion of OTUs, the data from edge-specific permutation and bootstrap score distributions with 1000 iterations were normalized; thus, the similarity introduced by only compositionality was acquired. Subsequently, a *P* value was obtained using pooled variance to z-score the permutated null and bootstrap confidence^64^. Network analyser cytoscape plug-in was used to calculate the topological parameters of the network. Gephi 1.9 was used to compute modularity and to visualize the network layout. A GLM (R package “MASS”) was used to test the centrality measures: degree of connection (extent of taxon connection working as hub) and closeness centrality (extent of influence of a network node), considering ‘Site’ (3 levels: Thuwal, Farasan, Mngazana) and ‘Burrow’ (2 levels: burrow, bulk) as explanatory variables. We used a negative binomial distribution family of the error, since the degree of connection is count data, while a quasipoisson distribution family was applied for closeness centrality. The function ‘varpart’ in the R package vegan^65^ was used to explore the variation explained by the three factors.

2-way ANOVA was used to test the difference in fluorescein levels between ‘Depth’ (levels: surface, subsurface, deep) and ‘Fraction’ (levels: 1, 2, 3, 4, 5, bulk). All statistical tests were performed using PRIMER v. 6.1, PERMANOVA+ for PRIMER routines and R software 3.4.1. All parametric tests met the assumptions of normality and homogeneity, or were transformed appropriately and non-parametric tests applied.

## Supplementary information

Supplementary Info

Supplementary Dataset S2

Supplementary Dataset S3

Supplementary Dataset S4

Supplementary File S5 (Video)

Supplementary File S6 (Video)

## Data Availability

Sequence data generated during the current study are available in the NCBI SRA repository under the BioProject ID: PRJNA339628.

## References

[1] Sloan WT (2006). Quantifying the roles of immigration and chance in shaping prokaryote community structure. Environ. Microbiol..

[2] Martiny JBH (2006). Microbial biogeography: Putting microorganisms on the map. Nat. Rev. Microbiol..

[3] Dumbrell AJ, Nelson M, Helgason T, Dytham C, Fitter AH (2010). Relative roles of niche and neutral processes in structuring a soil microbial community. ISME J..

[4] Tringe SG (2005). Comparative metagenomics of microbial communities. Science.

[5] Lennon JT, Aanderud ZT, Lehmkuhl BK, Schoolmaster DR (2012). Mapping the niche space of soil microorganisms using taxonomy and traits. Ecology.

[6] Valverde A, Makhalanyane TP, Cowan DA (2014). Contrasting assembly processes in a bacterial metacommunity along a desiccation gradient. Front. Microbiol..

[7] Falkowski PG, Fenchel T, Delong EF (2008). The microbial engines that drive Earth’s biogeochemical cycles. Science.

[8] Fierer N (2012). Cross-biome metagenomic analyses of soil microbial communities and their functional attributes. Proc. Natl. Acad. Sci..

[9] Lear G, Bellamy J, Case BS, Lee JE, Buckley HL (2014). Fine-scale spatial patterns in bacterial community composition and function within freshwater ponds. ISME J..

[10] Louca S (2016). High taxonomic variability despite stable functional structure across microbial communities. Nat. Ecol. Evol..

[11] Anantharaman K (2016). Thousands of microbial genomes shed light on interconnected biogeochemical processes in an aquifer system. Nat. Commun..

[12] Nelson MB, Martiny AC, Martiny JBH (2016). Global biogeography of microbial nitrogen-cycling traits in soil. Proc. Natl. Acad. Sci..

[13] Fernández A (1999). How stable is stable? Function versus community composition. Appl. Environ. Microbiol..

[14] Burke C, Steinberg P, Rusch DB, Kjelleberg S, Thomas T (2011). Bacterial community assembly based on functional genes rather than species. Proc. Natl. Acad. Sci..

[15] Louca S, Parfrey L, Doebeli M (2016). Decoupling function and taxonomy in the global ocean microbiome. Science.

[16] de Vries FT (2012). Abiotic drivers and plant traits explain landscape-scale patterns in soil microbial communities. Ecol. Lett..

[17] Ofijeru ID (2010). Combined niche and neutral effects in a microbial wastewater treatment community. Proc. Natl Acad. Sci..

[18] Elmqvist T (2003). Response diversity, ecosystem change, and resilience. Front. Ecol. Environ..

[19] Wellnitz T, Poff NLR (2001). Functional redundancy in heterogeneous environments: implications for conservation. Ecol. Lett..

[20] Aller, R. C. Benthic fauna and biogeochemical processes in marine sediments: the role of burrow structures in Nitrogen cycling in coastal marine environments (eds Blackburn, T. H. & Sorensen, J.) 301–338 (Wiley, 1988).

[21] Aller JY, Aller RC (1986). Evidence for localized enhancement of biological associated with tube and burrow structures in deep-sea sediments at the HEEBLE site, western NorthAtlantic. Deep Sea Res..

[22] Aller RC (1994). Bioturbation and remineralization of sedimentary organic matter: effects of redox oscillation. Chem. Geol..

[23] Mcclain ME (2003). Biogeochemical hot spots and hot moments at the interface of terrestrial and aquatic ecosystems. Ecosystems.

[24] Kuzyakov Y (2010). Priming effects: Interactions between living and dead organic matter. Soil Biol. Biochem..

[25] Vasquez-Cardenas D, Quintana CO, Meysman FJ, Kristensen E, Boschker TS (2016). Species-specific effects of two bioturbating polychaetes on sediment chemoautotrophic bacteria. Mar. Ecol. Prog. Ser..

[26] Papaspyrou S (2005). Sediment properties and bacterial community in burrows of the ghost shrimp P*estarella tyrrhena* (Decapoda: Thalassinidea). Aquat. Microb. Ecol..

[27] Kristensen E (2012). What is bioturbation? The need for a precise definition for fauna in aquatic sciences. Mar. Ecol. Prog. Ser..

[28] McClelland JW, Valiela I (1998). Linking nitrogen in estuarine producers to land-derived sources. Limnol. Oceanogr..

[29] Almahasheer H, Duarte CM, Irigoien X (2016). Nutrient limitation in central Red Sea mangroves. Front. Mar. Sci..

[30] Alvarenga DO, Rigonato J, Branco LHZ, Fiore MF (2015). Cyanobacteria in mangrove ecosystems. Biodivers. Conserv..

[31] Cuellar-Gempeler C, Leibold MA (2018). Multiple colonist pools shape fiddler crab-associated bacterial communities. ISME J..

[32] Shi S (2016). The interconnected rhizosphere: High network complexity dominates rhizosphere assemblages. Ecol. Lett..

[33] Ma B (2016). *G*eographic patterns of co-occurrence network topological features for soil microbiota at continental scale in eastern China. ISME J..

[34] Stams AJM, Plugge CM (2009). Electron transfer in syntrophic communities of anaerobic bacteria and archaea. Nat. Rev. Microbiol..

[35] Hall M (2010). Important plant areas in the Arabian Peninsula: 2. Farasan Archipelago. Edinburgh J. Bot..

[36] Varon-lopez M (2013). Sulphur-oxidizing and sulphate-reducing communities in Brazilian mangrove sediments. Environ. Microbiol..

[37] Fusi M (2015). Thermal specialization across large geographical scales predicts the resilience of mangrove crab populations to global warming. Oikos.

[38] Dittmann S (1996). Effects of macrobenthic burrows on infaunal communities in tropical tidal flats. Mar. Ecol. Prog. Ser..

[39] Andersen FO, Kristensen E (1988). Oxygen microgradients in the rhizosphere of the mangrove *Avicennia marina*. Mar. Ecol. Prog. Ser..

[40] Michaels RE, Zieman JC (2013). Fiddler crab (*Uca* spp.) burrows have little effect on surrounding sediment oxygen concentrations. J. Exp. Mar. Bio. Ecol..

[41] Konhauser, K. *Introduction to geomicrobiology*. (Blackwell publishing, 2007).

[42] Pülmanns N, Diele K, Mehlig U, Nordhaus I (2014). Burrows of the semi-terrestrial crab *Ucides cordatus* enhance CO_2_ release in a north brazilian mangrove forest. PLoS One.

[43] Bertics VJ, Ziebis W (2009). Biodiversity of benthic microbial communities in bioturbated coastal sediments is controlled by geochemical microniches. ISME J..

[44] Araújo JMC (2011). Selective geochemistry of iron in mangrove soils in a semiarid tropical climate: effects of the burrowing activity of the crabs *Ucides cordatus* and *Uca maracoani*. Geo-Marine Lett..

[45] Ding H, Yao S, Chen J (2014). Authigenic pyrite formation and re-oxidation as an indicator of an unsteady-state redox sedimentary environment: Evidence from the intertidal mangrove sediments of Hainan Island, China. Cont. Shelf Res..

[46] Kristensen E, Holmer M, Bussawarit N (1991). Benthic metabolism and sulfate reduction in a southeast Asian mangrove swamp. Mar. Ecol. Prog. Ser..

[47] Kristensen E, Alongi DM (2006). Control by fiddler crabs (*Uca vocans*) and plant roots (*Avicennia marina*) on carbon, iron, and sulfur biogeochemistry in mangrove sediment. Limnol. Oceanogr..

[48] Nielsen OI, Kristensen E, Macintosh DJ (2003). Impact of fiddler crabs (*Uca* spp.) on rates and pathways of benthic mineralization in deposited mangrove shrimp pond waste. J. Exp. Mar. Bio. Ecol..

[49] Burdorf LDW, Hidalgo-Martinez S, Cook PLM, Meysman FJR (2016). Long-distance electron transport by cable bacteria in mangrove sediments. Mar. Ecol. Prog. Ser..

[50] Nielsen LP, Risgaard-Petersen N, Fossing H, Christensen PB, Sayama M (2010). Electric currents couple spatially separated biogeochemical processes in marine sediment. Nature.

[51] Malkin SY (2014). Natural occurrence of microbial sulphur oxidation by long-range electron transport in the seafloor. ISME J..

[52] Hemmi JM, Zeil J (2003). Burrow surveillance in fiddler crabs II. The sensory cues. J. Exp. Biol..

[53] Reinsel A, Rittschof D (1995). Regulation of foraging in the sand fiddler crab *Uca pugilator* (Bosc 1802). J. Exp. Mar. Bio. Ecol..

[54] Andreetta A (2014). Mangrove carbon sink. Do burrowing crabs contribute to sediment carbon storage? Evidence from a Kenyan mangrove system. J. Sea Res..

[55] Kristensen E (2008). Mangrove crabs as ecosystem engineers; with emphasis on sediment processes. J. Sea Res..

[56] Walther GR, Berger S, Sykes MT (2005). An ecological ‘footprint’ of climate change. Proc. R. Soc. B Biol. Sci..

[57] Gattuso JP (2018). Ocean solutions to address climate change and its effects on marine ecosystems. Front. Mar. Sci..

[58] Finlay BJ, Maberly SC, Cooper JI (1997). Microbial diversity and ecosystem function. Oikos.

[59] Cordero OX, Datta MS (2016). Microbial interactions and community assembly at microscales. Curr. Opin. Microbiol..

[60] Moulton OM (2016). Microbial associations with macrobiota in coastal ecosystems: Patterns and implications for nitrogen cycling. Front. Ecol. Environ..

[61] Skov MW, Hartnoll RG (2002). Paradoxical selective feeding on a low-nutrient diet: Why do mangrove crabs eat leaves?. Oecologia.

[62] Schnurer J, Rosswall T (1982). Fluorescein diacetate hydrolysis as a measure of total microbial activity in soil and litter. Appl. Environ. Microbiol..

[63] Konishi, S. & Kitagawa, G. *Information criteria and statistical modeling*. (Springer Science & Business Media, 2008).

[64] Barberán A, Bates ST, Casamayor EO, Fierer N (2011). Using network analysis to explore co-occurrence patterns in soil microbial communities. ISME J..

[65] Oksanen J (2010). vegan: Community Ecology Package. R package version.

