# Supplementary material for: Fiddler crab bioturbation determines consistent changes in bacterial communities across contrasting environmental conditions
Source: Sci Rep. 2019 Mar 6;9:3749. doi: 10.1038/s41598-019-40315-0 (PMC6403291; doi:10.1038/s41598-019-40315-0)
Supplement: Supplementary file 1 — Supplementary Info [file 41598_2019_40315_MOESM1_ESM.docx]

**Supporting information**

**Supplementary File S1.** Supplementary Methods, Results, Discussion, Figures and Tables (Booth et al 2019_ Supplementary File S1.pdf)

**Supplementary File S2**. Mean values and standard error of the mean for geochemical variables and metals (values shown as ug mg^-1^) in Thuwal, Farasan and Mngazana (Booth et al 2019_ Supplementary File S2.xlsx)

**Supplementary File S3.** Linear discriminant analysis (Wilcoxon p-value: 0.05, LDA>2) Effect Size (LEfSe) of bacterial OTUs in bulk and burrow sediment in Thuwal, Farasan and Mngazana (Booth et al 2019_ Supplementary File S3.xlsx).

**Supplementary File S4.** Co-occurrence network analysis parameters (Booth et al 2019_ Supplementary File S4.xlsx)

**Supplementary File S5.** A fiddler crab plugs its burrow before the incoming tide. The crab is carving the mud plug that it will later use to block the entrance of its burrow (Booth et al 2019_ Supplementary File S5.mp4)

**Supplementary File S6.** A female fiddler crab restores its burrow during low tide, bringing mud pellets from inside the burrow to the surface. This process is likely responsible for the mixing of the bacterial communities from the deep with those of the surface (Booth et al 2019_ Supplementary File S6.mp4)

**Supplementary File 1: Supplementary Methods, Results, Discussion, Tables and Figures**

**Fiddler crab bioturbation determines consistent changes in bacterial communities across contrasting environmental conditions**

Jenny Marie Booth^†,^ Marco Fusi^†*^, Ramona Marasco, Tumeka Mbobo and Daniele Daffonchio^*^

^†^Co-first author

*Correspondence to:

[marco.fusi@kaust.edu.sa](mailto:marco.fusi@kaust.edu.sa)

[daniele.daffonchio@kaust.edu.sa](mailto:daniele.daffonchio@kaust.edu.sa)

**Methods**

**Biogeochemical, metal and grain size analysis.** All biogeochemical analyses of sediment were performed in GEOMAR (Kiel, Germany). Analysis of particulate carbon and nitrogen was undertaken by means of an elemental analyser (Thermo Finnegan Flash EA1112) using acetanilide as the calibration standard. POC (particulate organic carbon) and PON (particulate organic nitrogen) data were obtained through removal by acidification with sulphurous acid (H_2_SO_3_) under vacuum for 24-48 h, with subtraction from total carbon and nitrogen results yielding PIC (particulate inorganic carbon) and PIN (particulate inorganic nitrogen)^1^. For nutrient analysis, sediment was first leached with deionised water according to EPA protocols^2^. Nitrate, silicate and phosphate were analysed in sediment leaches using standard auto-analyser techniques on a Seal AA3 instrument, following the best practice guide for performing nutrient measurements^3^. Sulphate and chloride in the sediment leach solutions were measured using ion chromatography (Dionex)^4^. Sediment was dissolved using a microwave-assisted acid digestion procedure^5^ to produce a solution suitable for inductively coupled plasma mass spectrometry (ICP-MS) analysis. Subsequently, total elemental concentrations (U, Pb, Al, Mn, Fe, Co, Ti, Ni, V, and Cr) were obtained using an ICP-MS instrument (Element II, Thermo Fisher Scientific). Grain size measurements were obtained by laser diffractometry^6^.

**DNA extraction and metabarcoding analysis.** To identify the changes in bacterial community composition throughout burrow sediment profiles, we used 16S rRNA gene sequencing. DNA was extracted from a 0.4 g sub-sample of each 432 sediment samples using the Power Soil Total DNA Isolation Kit (MoBio Inc., CA, USA) following the manufacturer’s instructions. DNA was stained with Sybrsafe (Invitrogen™) and visualized by agarose gel (1%) electrophoresis before spectrophotometric quantification with Qubit™ 3.0 (Thermo Fisher Scientific). PCR amplification of the V4-V5 hypervariable regions of the 16S rRNA gene was performed using specific primers (341F, 785R) following a previously described protocol^7^.

**Raw data processing.** Raw forward and reverse reads for each sample were assembled into paired-end reads considering a minimum overlapping of 50 nucleotides and maximum one mismatch within the region using the fastq-join algorithm (https://code.google.com/p/ea-utils/wiki/FastqJoin), and quality checked to discard reads with mean quality below 20. UPARSE v8 and QIIME v1.8 software were used to dereplicate sequences. For reference chimera detection, the “Gold” database containing the chimera-checked reference database in the Broad Microbiome Utilities was used. Taxonomy was assigned in QIIME using UClust and the Greengenes database. After the exclusion of samples with low coverage (Good’s coverage value < 90), a total of 384 samples were obtained with 40, 530 OTUs overall belonging to the kingdom bacteria (⩾97% sequence similarity of 16S rRNA gene sequences). All analyses were performed on the filtered OTU table (reads filtered to 0.01%).

**Microbial activity.** Fluorescein diacetate (FDA) hydrolysis was used to assess total microbial hydrolysing activity in sediment^8^. This assay was restricted to Thuwal mangrove due to significant logistical constraints. Sediment was collected from six burrows following the design shown in Supplementary Fig. S8 and transported immediately to the laboratory. We added 1 g of sediment to 50 ml of sterile 60 mM sodium phosphate buffer (pH 7.6). 0.5 ml of 7.2 mM FDA substrate dissolved in acetone was then added, immediately mixed and incubated in the dark at 37 °C for 3 h on a rotary shaker (150 rpm). A control was also incubated (without sediment). As a further control, 1 g of sediment was autoclaved and the same procedure applied, to assess any fluorescence in the sediment not attributable to microorganism activity. After incubation, the reaction was terminated by the addition of 2 ml acetone and absorbance was read on a spectrophotometer at 490 nm. Fluorescein concentration released in each sample was calculated referring to curve previously calculated using a range of 0 µg to 5 µg fluorescein ml ^-1^ standards.

**Microsensor profiling.** Oxygen and redox (Eh) were measured with microsensors (UNISENSE, Aarhus, Denmark) in sediment cores extracted at low tide (during daylight) from Thuwal mangrove only, due to logistical constraints. Bioturbated sediment cores were taken around a central crab burrow using PVC cores (diameter 15 cm), while bulk sediment cores were taken in unbioturbated sediment as specified above. Profiling was performed immediately after core retrieval in the laboratory. An oxygen microsensor (Ox-200), with a tip diameter of 200 μm, was calibrated in sterile water at oxygen partial pressures of 0 and 21 kPa as previously described^9^. A Redox-200 microelectrode, with a tip diameter of 200 μm, was calibrated using two quinidrone solutions (10 mg ml^-1^) buffered at pH 4 and 7, respectively. Redox potential within the sediment was measured against an Ag-AgCl reference electrode provided by Unisense. A motorized micromanipulator was used to drive microsensors vertically into sediment cores at interval distances away from the burrow wall of 0.5, 1, 1.5, 3 and 4.5 cm (following the experimental design) and to a depth of 5 cm at a resolution of 200 μm. For each type of sensor, three replicates at each distance and for bulk sediment were performed. Signals from the microsensors were recorded directly on a laptop computer using the SensorsTrace Suite software (Unisense).

**Statistical analysis**

**Biogeochemical, metal and grain size analysis.** Due to the large number of biogeochemical and metal analytes, we first tested for multi-collinearity using the non-parametric Spearman correlation and Draftsman’s plots on normalized data prior. In cases where variables had a correlation coefficient higher than 0.85, we retained the most appropriate and informative variables. The biogeochemical analytes retained were POC, PON, PIN, PIC, nitrate, silicate, phosphate and sulphate and the metals retained were Fe, Pb and U.

**Results**

**Site characterization and environmental drivers shaping the bacterial community.**

Each sampled site shows a distinct physico-chemical properties A significant ‘Site’ × ‘Depth’ interaction was determined for grain size (PERMANOVA, *P* = 0.0047, Supplementary Table S1). Approximately 50% of dissimilarity was accounted for by a narrow range of grain sizes between Mngazana and Thuwal (53.2 to 63.6 µm) and Farasan and Thuwal (50.8 to 66.6 µm), and a larger range (13.72 to 424 µm) between Mngazana and Farasan (SIMPER).

A significant effect of ‘Site’ (*P* = 0.0001) was observed on metal content (3-way PERMANOVA, Supplementary Table S1). The metals Fe, Pb and U had different contributions to dissimilarity between sites. U contributed 80% to the dissimilarity between the two Saudi Arabian sites, with Fe contributing the remaining 20%. Instead, Pb and Fe combined to form a total of 75% dissimilarity between Mngazana and Thuwal and 88% between Mngazana and Farasan (SIMPER). All metals, Fe and Pb in particular, were more abundant in Mngazana sediment compared to the two Saudi Arabian sites (Supplementary File S2 online).

Community composition was significantly correlated with the metals Pb, Fe and U (DistLM, AICc = 1055.2, R^2^ = 0.46; Supplementary Fig. S2, Supplementary Table S2). Fe was a significant driver of bacterial community composition in Mngazana and Thuwal. Pb was also a significant driver of Mngazana sediment bacterial communities and of part of the Farasan community. Uranium particularly affected the bacterial communities in Farasan sediment.

Sediment bacterial community composition was significantly correlated with grain size (DistLM, AICc = 1020.2, R^2^ = 0.65; Supplementary Fig. S2, Supplementary Table S2). Small (0.73 and 0.354 µm), large (63.6, 76.2 µm) and intermediate grain size classes (e.g. 29.6, 22.6 µm) were significant drivers of community composition in Farasan, Thuwal, and Mngazana, respectively (supplementary Fig. S4, supplementary Table S3). Moreover, a significant interaction of ‘Depth’ × ‘Fraction’ was observed on the sediment phi value at each ‘Site’ (ANOVA; Thuwal: F_2,53_ = 4.48, *P* = 0.001; Farasan: F_10,47_ = 0.88, *P* = 0.0376; Mngazana: F_10,5_ = 2.01, *P* = 0.005).

**Discussion**

On a broad latitudinal scale, we studied three mangrove settings with contrasting ecological conditions: a riverine mangrove (Mngazana), a fringe mangrove (Thuwal) and an island coral archipelago fringe mangrove (Farasan). Riverine mangroves are characterized by strong outwelling from rivers, compared to bidirectional tidal flux in fringe mangroves^10^. Mangroves in riverine settings are known to be more efficient in nutrient cycling and better sinks for carbon, nitrogen and phosphorus than fringe mangroves^10^. At Mngazana, sediment was characteristic of a riverine mangrove, with high organic carbon and nitrogen input^11^, and silicate and phosphate in particular were enriched. This system also receives a significant input of organic nitrogen from the surrounding rural area where a large number of livestock are reared. Both of the Saudi Arabian sites were comparatively depleted in nutrients and metals, which is a typical characteristic of the Red Sea mangroves whose freshwater input is limited^11,12^. Consistent with the fossil coral bedrock of the island, Farasan sediment was characterized by higher levels of inorganic carbon than the other sites.

Rather than iron, lead and uranium were found to drive a portion of the bacterial community at Farasan. Although lead has previously been reported to be a significant trace metal in surface sediment in the region^13^, the levels we recorded were not high. In waterlogged sediment lead is known to be particularly complexed by organic acids^14^, and uranium adsorbs to organic matter in anoxic sediment^15^. Metal-reducing bacteria oxidise fermentative products of organic matter to reduce metals, and we found several bacteria known to reduce uranium (e.g. *Geobacter*, *Shewanella*, *Desulfovibrio* and *Pseudomonas*)^15^. Furthermore, metals can be directly precipitated by carbonates during decalcification^14^, which may be an explanation for the role of lead and uranium in driving bacterial community assemblage in Farasan.

Microbial abundance has been previously found to correlate with grain size in intertidal sediment^16^, and finer-grained sediment harbours higher bacterial abundance^17^. Here we show that bacterial community assemblage is also driven by grain size. This observation may be attributed to the effect of grain size on oxygen diffusion, pore water and on the fluxes of organic matter and minerals, with finer-grained sediment generally being enriched in organic carbon and lower dissolved oxygen concentrations, and fluxes of both being reduced in finer-grained sediment^18^.

**References**

1. Verardo, D. J., Froehlich, P. N. & McIntyre, A. Determination of organic carbon and nitrogen in marine sediments using the Carlo-Erba NA 1500 analyzer. *Deep Sea Res.* **37,** 157–165 (1990).

2. Plumb, R. *Procedures for handling and chemical analysis of sediment and water samples*. (State University of New York College at Buffalo Great Lakes Lab, 1981).

3. Grasshoff, K., Kremling, K. & Ehrhardt, M. *Methods of Seawater Analysis*. (Verlag Chemie, 1983).

4. Morales, A., Graterol, L. S. & Mesa, J. Determination of chloride, sulfate and nitrate in groundwater samples by ion chromatography. *Journal of Chromatography A* **884,** 185–190 (2000).

5. Hassan, N. M., Rasmussen, P. E., Dabek-Zlotorzynska, E., Celo, V. & Chen, H. Analysis of environmental samples using microwave-assisted acid digestion and inductively coupled plasma mass spectrometry: maximizing total element recoveries. *Water, Air, Soil Pollut.* **178**, 323–334 (2007).

6. Beuselinck, L., Govers, G., Poesen, J., Degraer, G. & Froyen, L. Grain-size analysis by laser diffractometry: Comparison with the sieve-pipette method. *Catena* **32,** 193–208 (1998).

7. Quast, C. *et al.* Evaluation of general 16S ribosomal RNA gene PCR primers for classical and next-generation sequencing-based diversity studies. *Nucleic Acids Research* **41,** 1–11 (2013).

8. Schnurer, J. & Rosswall, T. Fluorescein diacetate hydrolysis as a measure of total microbial activity in soil and litter. *Appl. Environ. Microbiol.* **43,** 1256–1261 (1982).

9. Bertics, V. J. & Ziebis, W. Biodiversity of benthic microbial communities in bioturbated coastal sediments is controlled by geochemical microniches. *ISME J.* **3,** 1269–85 (2009).

10. Ewel, K. C., Twilley, R. R. & Ong, J. E. Different kinds of mangrove forests provide different goods and services. *Glob. Ecol. Biogeogr. Lett.* **7,** 83–94 (1998).

11. Duarte, C. M. *et al.* Relationship between sediment conditions and mangrove *Rhizophora apiculata* seedling growth and nutrient status. *Mar. Ecol. Prog. Ser.* **175,** 277–283 (1998).

12. Almahasheer, H., Duarte, C. M. & Irigoien, X. Nutrient limitation in central Red Sea mangroves. *Front. Mar. Sci.* **3,** 1–14 (2016).

13. Usman, A. R. A., Alkredaa, R. S. & Al-wabel, M. I. Ecotoxicology and environmental safety heavy metal contamination in sediments and mangroves from the coast of Red Sea: *Avicennia marina* as potential metal bioaccumulator. *Ecotoxicol. Environ. Saf.* **97,** 263–270 (2013).

14. Charlatchka, R. & Cambier, P. Influence of reducing conditions on solubility of trace metals in contaminated soils. *Water, Air, Soil Pollut.* **118,** 143–167 (2000).

15. Wall, J. D. & Krumholz, L. R. Uranium Reduction. *Annu. Rev. Microbiol.* **60,** 149–166 (2006).

16. Dale, N. C. Bacteria in intertidal sediments: Factors related to their distribution. *Limnol. Oceanogr.* **19,** 509–518 (1974).

17. Yamamoto, N. & Lopez, G. Bacterial abundance in relation to surface area and organic content of marine sediments. *J. Exp. Mar. Bio. Ecol.* **90,** 209–220 (1985).

18. Tyson, R. V. Abundance of organic matter in sediments in Sedimentary Organic Matter 81–118 (Springer, 1991).

19. Ghazanfar, S. A. & Fisher, M. *Vegetation of the Arabian peninsula. Vol. 25*. (Springer Science & Business Media, 2013).

20. Rasul, M. A. & Stewart, C. F. *The Red Sea: The formation, morphology, oceanography and environment of a young ocean basin* (Springer Earth System Sciences, 2015).

21. Mandura, A. S., Saifullah, S. M. & Khafaji, A. K. Mangrove ecosystem of southern Red Sea coast of Saudi Arabia. *Proc. Saudi Biol. Soc.* **10,** 165–193 (1987).

22. Gladstone, W. *Farasan Islands Protected Area master management plan*. (NCWCD, 2000).

23. Edgell, H. S. Coastal deserts of Arabia. in *Arabian Deserts: Nature, Origin, and Evolution* 385–426 (Springer Science & Business Media, 2006).

24. Adame, M. F. & Lovelock, C. E. Carbon and nutrient exchange of mangrove forests with the coastal ocean. *Hydrobiologia* **663,** 23–50 (2011).

25. Fusi, M. *et al.* Thermal specialization across large geographical scales predicts the resilience of mangrove crab populations to global warming. *Oikos* **124,** 784–795 (2015).

26. Rajkaran, A. & Adams, J. The effects of environmental variables on mortality and growth of mangroves at Mngazana Estuary, Eastern Cape, South Africa. *Wetl. Ecol. Manag.* **20,** 297–312 (2012).

**Supplementary Figures**


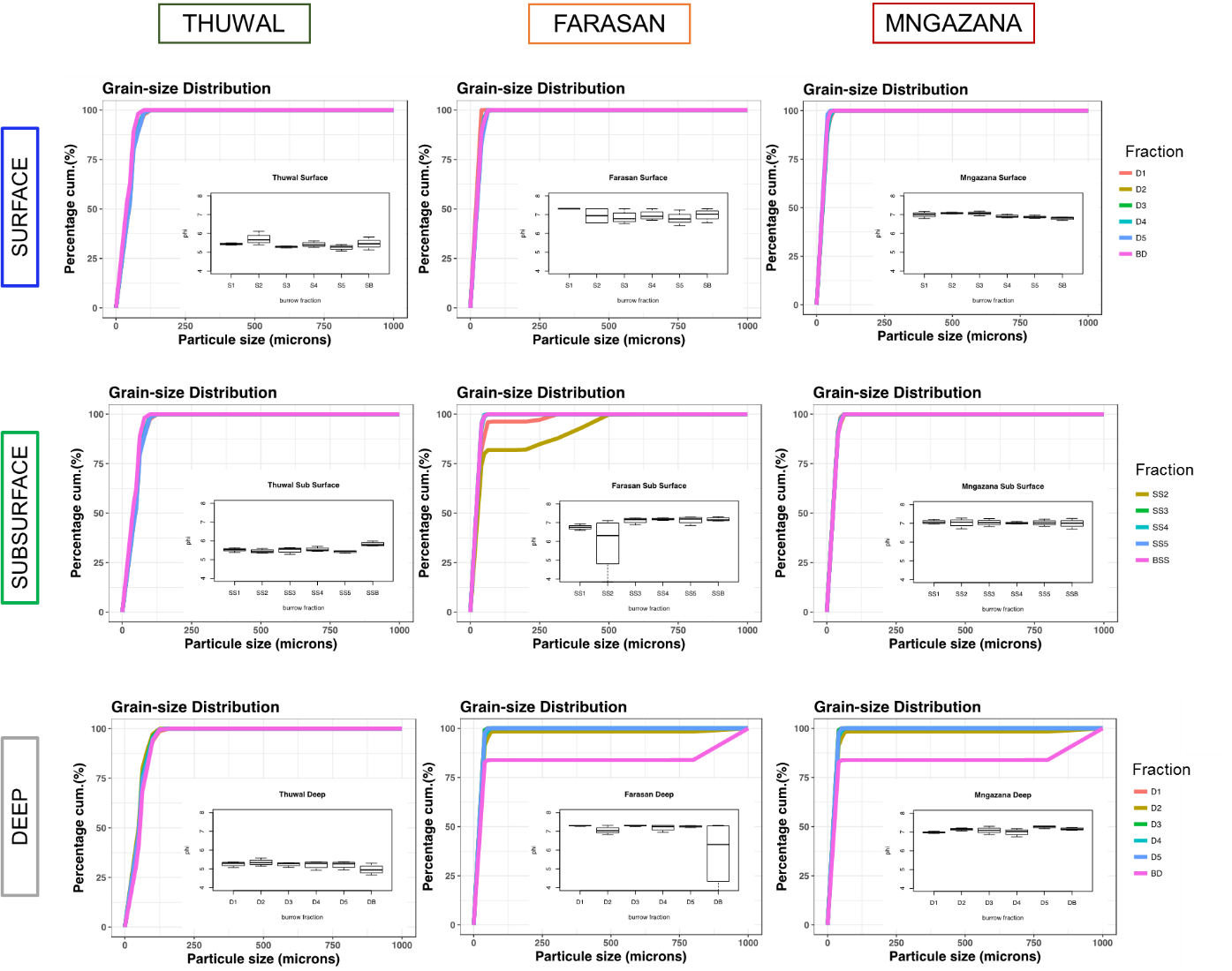


**Fig. S1. Grain size distribution.** Grain size distribution at each site and depth are shown, with cumulative percentage on the y-axis. Boxplots show the median phi for each site and depth, which summarizes the overall grainsize contribution. Sediment fraction is shown on the x-axis and the median phi on the y axis. In each plot n = 18.

**Fig. S2. Distance-based redundancy analysis (db-RDA) on sediment environment drivers of bacterial communities.** Db-RDA of **(a)** metal content and **(b)** grain size at each site, showing the main drivers of bacterial community composition (n = 54 for each site). Values in **(b)** show grain size classes (μm).


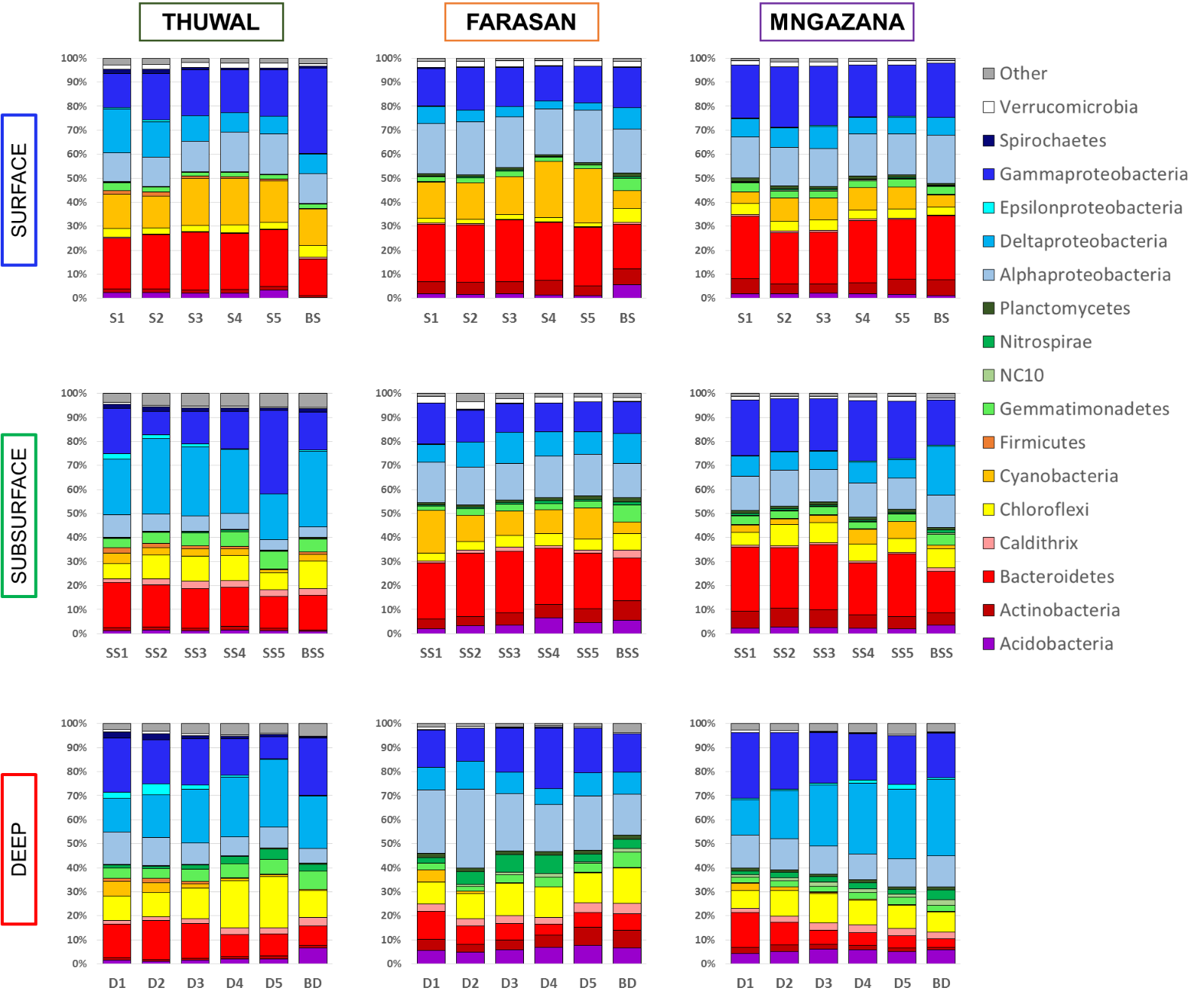


**Fig. S3. Taxonomic classification of sediment fraction bacterial communities.** Phylum-level taxonomic classification of sediment communities (OTU ≥ 97%) based on 16S rRNA gene sequencing. Phyla classified within Other (with less than 1% contribution to total community composition in at least one sample): AC1, *Betaproteobacteria*, BRC1, *Chlorobi*, *Elusimicrobia*, *Fusobacteria*, GN02, GN04, *Lentisphaerae*, NC10, NKB19, OD1, OP1, OP8, SAR406, SBR1093, *Tenericutes*, *Thermi*, TM6, TM7, WS3 and *Zetaproteobacteria*.


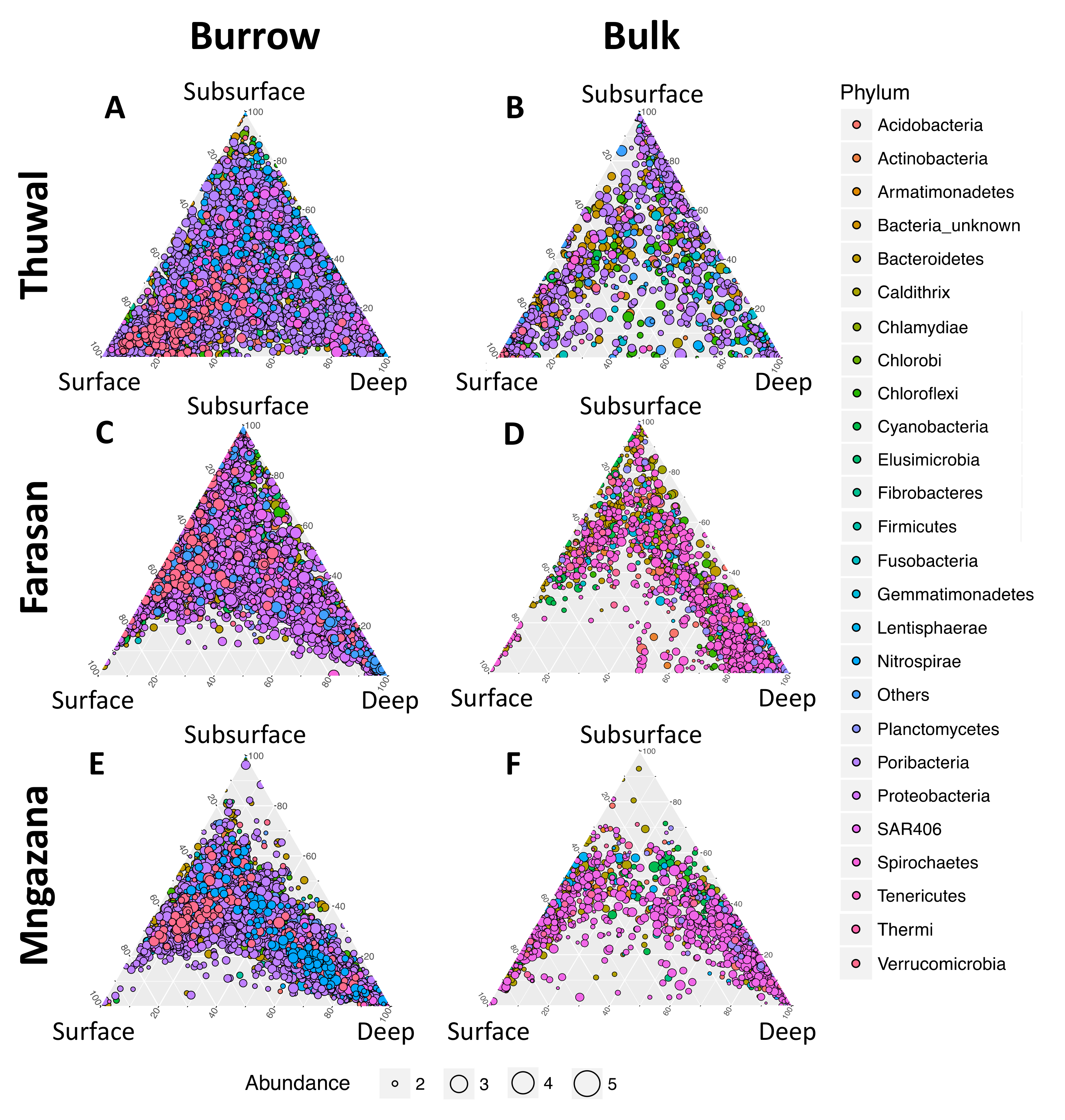


**Fig. S4.** **Ternary plots of OTU sharing among sediment depths.** Ternary plots of all bacterial OTUs detected (with relative abundance > 0.01% in at least one sample) in burrow and bulk sediment at **(A,B)** Thuwal, **(C,D)** Farasan and **(E,F)** Mngazana. Relative abundance of each OTU (log transformed - weighted average) is represented by each circle to show the contribution of each phyla in the surface, subsurface and deep communities to total relative abundance. Circle size is proportional to mean abundance within the total sediment community. Axes indicate the contribution of each depth level. Phyla are represented by colour code.

**Fig. S5. Linear discriminant analysis effect size (LEfSe) displaying differences in sediment microbial community taxonomic composition.** Cladograms indicating the taxonomic distribution of taxonomic lineages statistically different between burrow and bulk sediment at Thuwal, Farasan and Mngazana. LDA scores determined by LEfSe (>2.0) are displayed. Lineages with no taxa significantly enriched in either burrow or bulk are denoted with a yellow circle. Red taxa are significantly more abundant in bulk sediment and green taxa significantly more enriched in burrow sediment.

**Fig. S6. Boxplots of counts of bacterial OTUs assigned to a predicted function in the sediment of Thuwal, Farasan and Mngazana mangroves.** Bacterial OTUs from burrow sediment are shown in green and those from bulk sediment are shown in red. **(A)** aerobic nitrite oxidation, **(B)** Denitrification, **(C)** Nitrogen fixation, **(D)** Cyanobacteria, **(E)** Anoxygenic photoautotrophy, **(F)** Oxygenic photoautotrophy, **(G)** Photoheterotrophy, **(H)** Phototrophy, **(I)** Chemoheterotrophy.

**
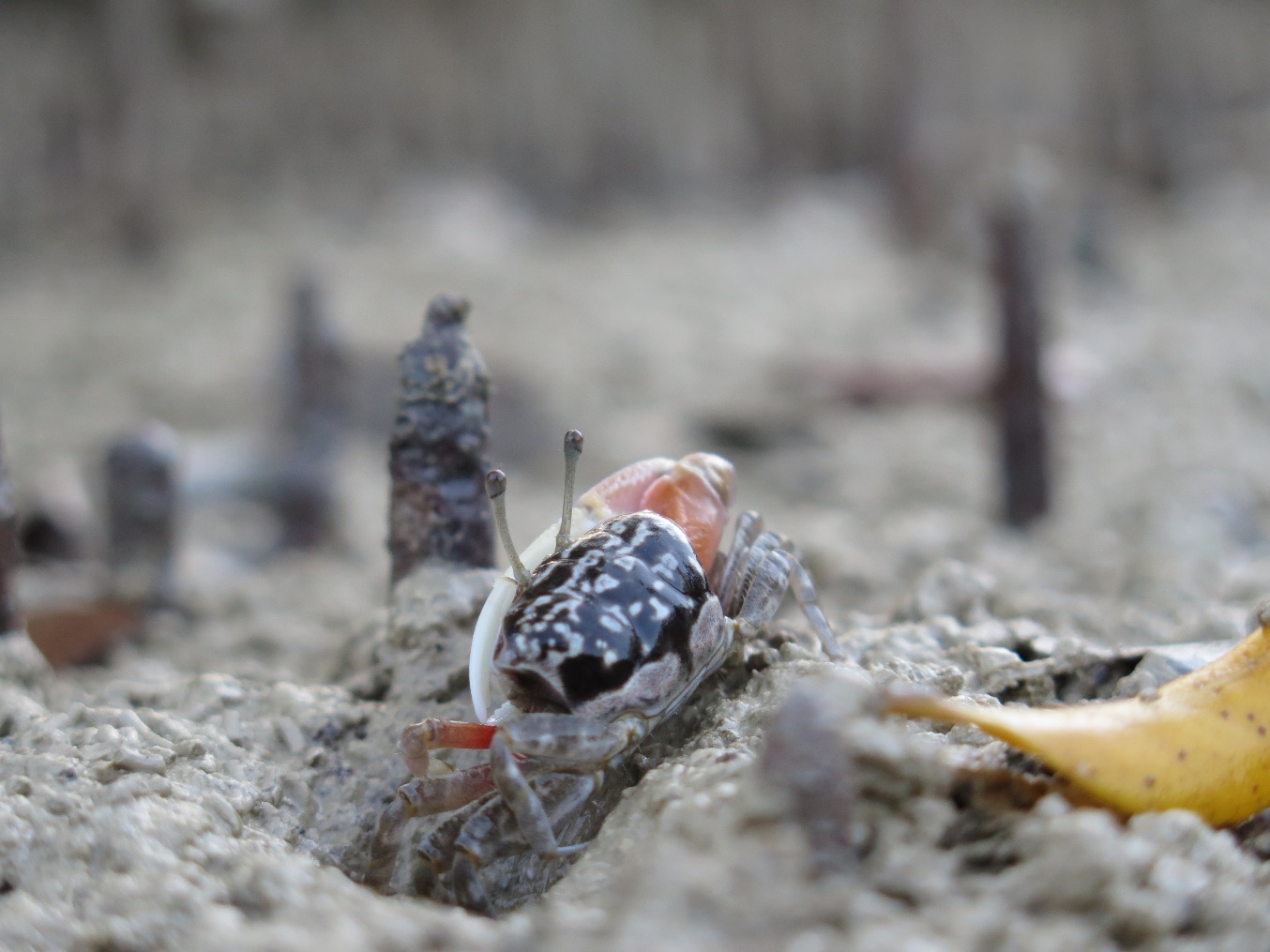
**

**Fig. S7. Study species: *Cranuca inversa* (male)**. Male specimen of ***Cranuca inversa*** at the burrow entrance in Farasan Island mangrove forest, Saudi Arabia.


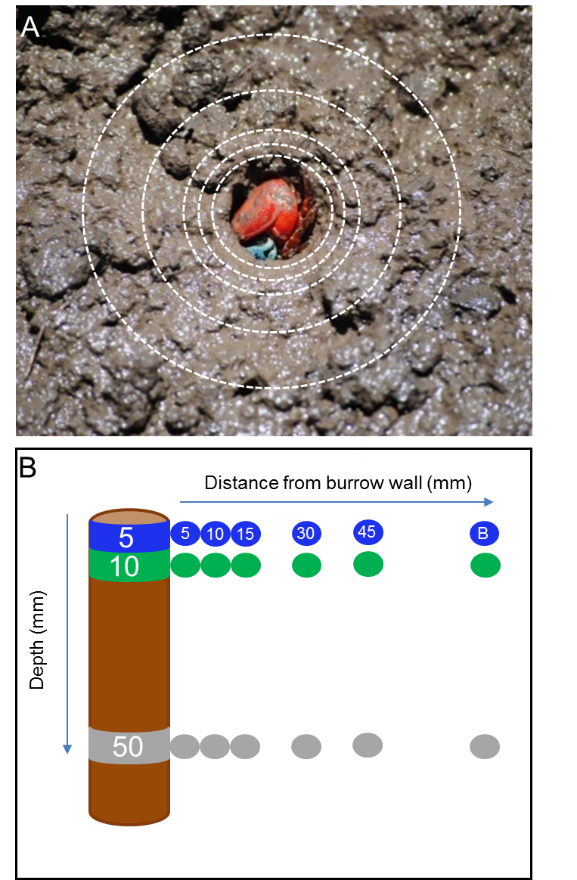


**Fig. S8**. **Experimental design.** **(a)** Photograph of a fiddler crab burrow displaying an overlay of the radial sampling design. **(b)** Detailed schematic of sediment sampling design in both the horizontal and vertical direction (B: bulk sediment).

**Table S1**. 3-way PERMANOVA to test effect of ‘Site’, ‘Depth’ and ‘Burrow’ on **(A)** Biogeochemistry, **(B)** metal content and **(C)** grain size. ‘Site’ (3 levels: Thuwal, Farasan, Mngazana); ‘Depth’ (3 levels: surface, subsurface, deep); ‘Burrow’ (2 levels: bulk, burrow). Significant effects and interactions are highlighted in bold. Df: degrees of freedom; MS: mean sum of squares; Pseudo-F: F value by permutation; P: p statistic.

| 1. **Biogeochemistry** |  |  |  |  |
| --- | --- | --- | --- | --- |
| **Source** | **df** | **MS** | **Pseudo-F** | ***P*** |
| Site | 2 | 381.7 | 89.964 | **0.0001** |
| Depth | 2 | 7.588 | 1.7887 | 0.0978 |
| Burrow | 1 | 16.21 | 3.82 | **0.0138** |
| Site × Depth | 4 | 4.942 | 1.1649 | 0.2786 |
| Site × Burrow | 2 | 7.408 | 1.7462 | 0.1004 |
| Depth × Burrow | 2 | 2.465 | 0.58111 | 0.7635 |
| Site × Depth × Burrow | 4 | 0.978 | 0.23057 | 0.9997 |
| Res | 144 | 4.242 |  |  |
| Total | 161 |  |  |  |
|  |  |  |  |  |
| **(B) Metals** |  |  |  |  |
| **Source** | **df** | **MS** | **Pseudo-F** | ***P*** |
| Site | 2 | 106.4 | 60.49 | **0.0001** |
| Depth | 2 | 0.827 | 0.47023 | 0.753 |
| Burrow | 1 | 3.303 | 1.8786 | 0.154 |
| Site × Depth | 4 | 1.966 | 1.118 | 0.3489 |
| Site × Burrow | 2 | 1.15 | 0.6542 | 0.6205 |
| Depth × Burrow | 2 | 0.121 | 6.86E-02 | 0.9937 |
| Site × Depth × Burrow | 4 | 0.13 | 7.42E-02 | 0.9999 |
| Res | 143 | 1.758 |  |  |
| Total | 160 |  |  |  |
|  |  |  |  |  |
| **(C) Grain size** |  |  |  |  |
| **Source** | **df** | **MS** | **Pseudo-F** | ***P*** |
| Site | 2 | 3046 | 185.27 | **0.0001** |
| Depth | 2 | 45.91 | 2.7928 | **0.0311** |
| Burrow | 1 | 8.053 | 0.48987 | 0.6228 |
| Site × Depth | 4 | 49.47 | 3.0093 | **0.0047** |
| Site × Burrow | 2 | 19.64 | 1.1944 | 0.2943 |
| Depth × Burrow | 2 | 11.81 | 0.71864 | 0.5614 |
| Site × Depth × Burrow | 4 | 13.33 | 0.81084 | 0.5748 |
| Res | 134 | 16.44 |  |  |
| Total | 151 |  |  |  |

**Table S2**. DistLM showing main contributing drivers of microbial community assemblage in terms of (A) biogeochemistry, (B) metal content and (C) grain size. Explanatory variables are shown in the first column. AICc: Akaike Information Criterion value; SS: Sum of square; Pseudo-F: F statistic; *P*: p statistic; Cumul.: cumulative variance explained; res.df: residual degree of freedom.

| **(A) Biogeochemistry** | | |  |  |  |  |  |
| --- | --- | --- | --- | --- | --- | --- | --- |
| **Variable** | **AICc** | | **SS(trace)** | **Pseudo-F** | ***P*** | **Cumul.** | **res.df** |
| POC (+) | | 1154.8 | 92008 | 74.733 | 0.0001 | 0.3184 | 160 |
| PON (+) | | 1144.8 | 14183 | 12.336 | 0.0001 | 0.3675 | 159 |
| PIN (+) | | 1140.3 | 7233.2 | 6.5094 | 0.0003 | 0.3925 | 158 |
| Nitrate (+) | | 1135.5 | 7444.8 | 6.9522 | 0.0002 | 0.4182 | 157 |
| Nitrite (+) | | 1130.7 | 6973.1 | 6.7502 | 0.0002 | 0.4424 | 156 |
| Silicate (+) | | 1129.2 | 3672.9 | 3.6151 | 0.0052 | 0.4551 | 155 |
|  |  | |  |  |  |  |  |
| **(B) Metal content** | | |  |  |  |  |  |
| **Variable** | **AICc** | | **SS(trace)** | **Pseudo-F** | ***P*** | **Cumul.** | **res.df** |
| Pb (+) | 1083.3 | | 77099 | 78.72 | 0.001 | 0.3368 | 155 |
| Fe (+) | 1055.5 | | 26324 | 32.306 | 0.001 | 0.4518 | 154 |
| U (+) | 1055.2 | | 1940.6 | 2.4033 | 0.031 | 0.4603 | 153 |
|  |  | |  |  |  |  |  |
| **(C) Grain size** | | |  |  |  |  |  |
| **Variable** | **AICc** | | **SS(trace)** | **Pseudo-F** | ***P*** | **Cumul.** | **res.df** |
| 63.6 | 1084.9 | | 99259 | 83.775 | 0.0001 | 0.3568 | 151 |
| 0.354 | 1042.5 | | 45158 | 50.644 | 0.0001 | 0.5192 | 150 |
| 29.6 | 1039.3 | | 4550.4 | 5.2478 | 0.0002 | 0.5355 | 149 |
| 76.2 | 1036.5 | | 4105.4 | 4.8571 | 0.0002 | 0.5503 | 148 |
| 2.96 | 1034 | | 3764.2 | 4.5605 | 0.0002 | 0.5638 | 147 |
| 44.4 | 1032.3 | | 3070.6 | 3.7908 | 0.0007 | 0.5749 | 146 |
| 236 | 1031.8 | | 2018.3 | 2.5176 | 0.0366 | 0.5821 | 145 |
| 10 | 1031.3 | | 2148.9 | 2.7122 | 0.0081 | 0.5898 | 144 |
| 7.62 | 1028.2 | | 3916.5 | 5.0833 | 0.0001 | 0.6039 | 143 |
| 22.6 | 1027.1 | | 2402.3 | 3.1652 | 0.0017 | 0.6126 | 142 |
| 2.36 | 1026.6 | | 1929.4 | 2.5504 | 0.0103 | 0.6138 | 142 |
| 0.73 | 1023.7 | | 3652.5 | 4.9629 | 0.0002 | 0.627 | 141 |
| 1.254 | 1020.4 | | 3368.8 | 4.6786 | 0.0002 | 0.635 | 141 |
| 15.7 | 1020.3 | | 1649.5 | 2.3121 | 0.0173 | 0.641 | 140 |
| 114.4 | 1020.2 | | 1605.6 | 2.2711 | 0.0172 | 0.6467 | 139 |

**Table** **S3**. Canonical Analysis of Principal components cross-validation table of OTU assemblages for each fraction at different sites and depths. Site is the sampling location, Depth is the depth of sampling of all the fractions (Figure S4), Choice of m is the choice of the minimum number of axes based on the minimum misclassification error or minimum residual sum of squares, delta_1^2 is the first squared canonical correlation and *P* is the p statistic.

| **Site** | **Depth** | **Choice of m** | **delta_1^2** | ***P*** |
| --- | --- | --- | --- | --- |
| Thuwal | Surface | 28 | 0.97043 | 0.0038 |
|  | Subsurface | 26 | 0.89334 | 0.0286 |
|  | Deep | 12 | 0.76537 | 0.0044 |
| Farasan | Surface | 28 | 0.9791 | 0.0029 |
|  | Subsurface | 21 | 0.90039 | 0.0035 |
|  | Deep | 12 | 0.8325 | 0.0009 |
| Mngazana | Surface | 18 | 0.96574 | 0.0047 |
|  | Subsurface | 16 | 0.82229 | 0.0293 |
|  | Deep | 16 | 0.9687 | 0.0001 |

**Table** **S4** - Network topological coefficients.

| **Location** | **Zone** | **Modularity** | **Clustering Coefficient** | **Network Centralization** | **Density** | **Interactions** | **Mutual Exclusion** | **Co-presence** |
| --- | --- | --- | --- | --- | --- | --- | --- | --- |
| Thuwal | Bulk | 0.681 | 0.281 | 0.143 | 0.05 | 1583 | 394 | 1189 |
| Thuwal | Burrow | 0.754 | 0.335 | 0.315 | 0.053 | 2114 | 694 | 1420 |
| Farasan | Bulk | 0.601 | 0.108 | 0.16 | 0.07 | 153 | 27 | 126 |
| Farasan | Burrow | 0.562 | 0.072 | 0.14 | 0.06 | 134 | 23 | 111 |
| Mngazana | Bulk | 0.49 | 0.275 | 0.153 | 0.032 | 2655 | 1049 | 1606 |
| Mngazana | Burrow | 0.503 | 0.34 | 0.26 | 0.048 | 3039 | 1305 | 1734 |

**Table** **S5** - 3-way PERMANOVA to test effect of ‘Site’, ‘Depth’ and ‘Burrow’ on the predicted function of the bacterial communities. ‘Site’ (3 levels: Thuwal, Farasan, Mngazana); ‘Depth’ (3 levels: surface, subsurface, deep); ‘Burrow’ (2 levels: bulk, burrow). Significant effects and interactions are highlighted in bold. Df: degrees of freedom; MS: mean sum of squares; Pseudo-F: F value by permutation; P: p statistic.

| **Source** | **df** | **MS** | **F** | ***P*** |
| --- | --- | --- | --- | --- |
| Site | 2 | 24673 | 17.36 | **0.001** |
| Depth | 2 | 16968 | 11.939 | **0.001** |
| Burrow | 1 | 3812.9 | 2.6829 | **0.032** |
| Site × Depth | 4 | 5265.6 | 3.705 | **0.001** |
| Site × Burrow | 2 | 1974.9 | 1.3896 | 0.194 |
| Burrow × Depth | 2 | 2242 | 1.5775 | 0.12 |
| Site × Depth × Burrow | 4 | 1430.3 | 1.0064 | 0.44 |
| Res | 366 | 1421.2 |  |  |

**Table** **S6.** Description of main environmental characteristics of each sampling site.

| **Location** | **Co-ordinates** | **Average temp. (° C)** | **Average precipitation (mm)** | **Tidal range (m)** | **Geomorphological setting** | **Sediment composition** | **Dominant mangrove tree species** | **Source** |
| --- | --- | --- | --- | --- | --- | --- | --- | --- |
| *Thuwal,*  *Saudi Arabia* | 22°33'N 41°24'E | 29.8 | 7.6 | 0.3 | Mainland mangrove on desert coastline | > 98% sand | *Avicennia marina* | ^19,20^ |
| *Farasan Island, Saudi Arabia* | 16°20'N 41°24'E | 31 | 50 | 0.4 | Fossil Coral Reef originated island | > 98% sand | *Avicennia marina, Rhizophora mucronata* | ^21–23^ |
| *Mngazana,*  *South Africa* | 31°42'S 29°25E | 18 | 1200 | 2.5 | River-dominates, mangroves form on deltaic islands at river mouth | fine sediment mainly composed by silt and clay | *Avicennia marina, Rhizophora mucronata, Bruguiera spp.* | ^24–26^ |
